# Treatment of hydroxyurea-resistant/intolerant polycythemia vera: a discussion of best practices

**DOI:** 10.1007/s00277-023-05172-y

**Published:** 2023-03-21

**Authors:** Andrew T. Kuykendall

**Affiliations:** Department of Malignant Hematology, H. Lee Moffitt Cancer Center, Tampa, FL 33612 USA

**Keywords:** Polycythemia vera, Ruxolitinib, Myeloproliferative neoplasm, Janus kinase

## Abstract

Polycythemia vera (PV) is a burdensome, chronic myeloproliferative neoplasm characterized by activating mutations in Janus kinase 2, erythrocytosis, and bone marrow hypercellularity. The goals of treatment are to achieve hematocrit and blood count control to ultimately reduce the risk of thrombohemorrhagic events and improve PV-related symptoms. Treatment is risk-stratified and typically includes cytoreduction with hydroxyurea or interferon formulations in first line for high-risk disease. However, inadequate response, resistance, or intolerance to first-line cytoreductive therapies may warrant introduction of second-line treatments, such as ruxolitinib. In this review, I detail preferred treatment and patient management approaches following inadequate response to or intolerance of first-line treatment for PV.

## Introduction 

Polycythemia vera (PV) is a chronic myeloproliferative neoplasm characterized by erythrocytosis, bone marrow hypercellularity, and activating Janus kinase (JAK) mutations (*JAK2*V617F or *JAK2* exon 12 mutations) that is estimated to affect more than 100,000 people in the USA [1, 2]. Patients with PV have burdensome signs and symptoms including pruritus, fatigue, night sweats, concentration problems, and splenomegaly [3, 4]. There are also increased risks of thrombosis, mortality, and disease transformation to myelofibrosis or acute myeloid leukemia [5–10].

Polycythemia vera treatment goals include controlling blood counts (hematocrit, < 45%; white blood cell [WBC] count, < 11 × 10^9^/L; platelet count, < 400 × 10^9^/L), resolving disease-related signs and symptoms, and reducing risk of thromboembolic events [11–13]. Hydroxyurea (HU) and interferon formulations are recommended first-line treatments for patients with high-risk PV (age ≥ 60 years or history of thrombosis). However, up to 25% of patients become resistant to or intolerant of HU, and historically, interferon therapy has been associated with challenging side effects [14–17]. Furthermore, although HU is effective at reducing thromboses in patients with PV [16, 18], HU treatment has not generally been shown to improve symptoms or modify the disease [19–21]. Ruxolitinib, a JAK1 and JAK2 inhibitor, provides clinical benefit, including hematocrit and WBC count control and reductions in splenomegaly and symptom burden, and was approved by the US Food and Drug Administration in 2014 for PV in adults who have an inadequate response to or are intolerant of HU [22–29].

### Sample patient 1 — part 1

A 32-year-old male patient presented to urgent care with persistent left upper quadrant abdominal pain for the previous 4 weeks. Computed tomography scan showed a 15 × 12 × 12.5 cm spleen with multiple splenic infarcts. Laboratory work showed that hemoglobin was 19.8 g/dL, and hematocrit was 60.3%. WBC count was 10.9 × 10^9^/L, platelets were 276 × 10^9^/L, lactose dehydrogenase was 696 units/L, ferritin was 13.5 μg/L, and erythropoietin was 1 mIU/mL. *JAK2* quantitative polymerase chain reaction showed presence of the *JAK2*V617F mutation with allele frequency of 36%. Bone marrow biopsy showed hypercellular marrow with erythroid hyperplasia and no increase in iron stores. Karyotype was normal. The patient was started on aspirin 81 mg daily and phlebotomy with a goal hematocrit < 45%.

### Sample patient 2 — part 1

A 50-year-old female patient presented with debilitating migraines with visual auras. Blood counts were as follows: hemoglobin, 19.5 g/dL; hematocrit, 58.6%; WBC, 11.4 × 10^9^/L; and platelets, 532 × 10^9^/L. *JAK2*V617F was qualitatively positive. Bone marrow biopsy was consistent with PV without fibrosis. The patient was started on aspirin 81 mg daily and HU.

## Treatment goals and first-line treatment options in PV

The primary treatment goals in my practice are to reduce the risk of thrombosis and hemorrhage and maintain blood count control [11–13, 30, 31]. We balance that with a comprehensive patient management approach that aims to improve symptom burden and quality of life (QoL) in patients with PV.

**Fig. 1:**
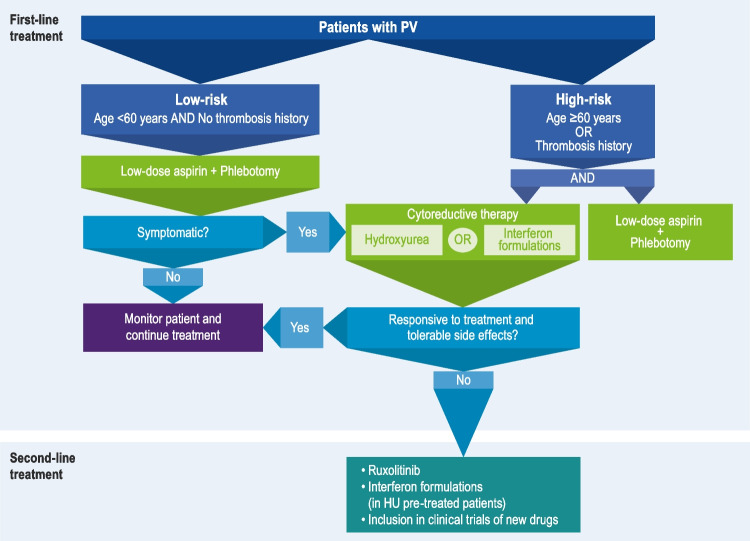
Therapeutic strategy for polycythemia vera. PV, polycythemia vera

Treatment for patients with PV should be guided by the patient’s risk status (Fig. 1). Patients with low-risk disease (age < 60 years and no history of thrombosis) are treated with low-dose aspirin and therapeutic phlebotomy to maintain hematocrit < 45%. For patients with high-risk disease, the addition of cytoreductive therapy is recommended. Specifically, HU, pegylated interferon alfa-2a, or ropeginterferon alfa-2b may be considered, which have been shown to have similar clinical benefit in the treatment of PV, despite varying mechanisms of action, modes of administration, and toxicity profiles [16, 30, 32–35].

HU, a ribonucleotide reductase inhibitor, has traditionally been the standard cytoreductive agent administered to high-risk patients with PV [36]. However, a considerable number of patients are either intolerant to HU or are HU resistant [14–17, 33]. Various interferon formulations have also been widely used in patients with PV for decades, given their antiproliferative, immunomodulating, and anticlonal effects [17]. More recently, pegylated forms of interferon, specifically pegylated interferon alfa-2a (an interferon with six monopegylated positional isomers) and ropeginterferon alfa-2b (a monopegylated interferon with a single positional isomer associated with an extended elimination half-life), have been recommended for first-line PV treatment due to their longer acting effects, less frequent administration, and tolerable side effects [16, 32, 33, 37, 38].

### Sample patient 1—part 2

Three years after diagnosis, the patient experienced rapidly increasing spleen size with associated abdominal discomfort and unintentional weight loss of 11 kg. Laboratory work showed that hemoglobin was 14.6 g/dL, hematocrit was 45%, and WBC count was 19 × 10^9^/L. A repeat bone marrow biopsy was performed, which showed 100% cellularity with panmyelosis (trilineage hyperplasia), megakaryocyte clustering, and mild reticulin fibrosis. Karyotype showed 47, XY, + 9 [11] /47, XY, del(20q)(q11.2q13.2) [9], suggesting cytogenetic evolution with uniparental disomy. Next-generation sequencing showed variant allele fraction of *JAK2*V617F mutation to be 63%, corresponding with an additional copy of 9th chromosome.

### Sample patient 2—part 2

Treatment with first-line HU at 1000 mg daily for 6 months was associated with improvements in the patient’s headaches but led to intolerable mouth ulcers and hair loss.

## Optimal time to proceed to second-line treatment

In my practice, we monitor for several factors that indicate patients may benefit from a change in treatment. These include new thrombosis or disease-related major bleeding, frequent phlebotomy or phlebotomy intolerance, splenomegaly, progressive thrombocytosis and/or leukocytosis, or new or worsening disease-related symptoms (e.g., pruritus, night sweats, fatigue) [16]. For HU specifically, European LeukemiaNet established a consensus definition for resistance or intolerance that my practice follows [15]. These criteria define HU resistance or intolerance in PV as one or more of the following: (a) need for phlebotomy to maintain hematocrit < 45% after 3 months of ≥ 2 g/day HU, (b) uncontrolled myeloproliferation (i.e., platelet count > 400 × 10^9^/L and WBC count > 10 × 10^9^/L) after 3 months of ≥ 2 g/day HU, (c) failure to reduce massive splenomegaly (i.e., organ extending by > 10 cm from costal margin) by > 50% as measured by palpation or failure to completely relieve symptoms related to splenomegaly after 3 months of ≥ 2 g/day HU, (d) absolute neutrophil count < 1.0 × 10^9^/L, platelet count < 100 × 10^9^/L, or hemoglobin < 100 g/L at the lowest dose of HU required to achieve a complete or partial response, or (e) the presence of leg ulcers or other unacceptable HU-related nonhematologic toxicities (e.g., mucocutaneous manifestations, gastrointestinal symptoms, pneumonitis, or fever at any dose of HU). It is important to note that 2 g/day HU is often difficult to tolerate, in which case our practice evaluates patients using these same criteria at the patient’s maximum tolerated dose. We also recommend hematocrit monitoring at each phlebotomy or at least every 3 months to ensure levels < 45%. There are no formal guidelines that define inadequate response or intolerance for the various available interferon formulations. In my practice, emergence of new or worsening PV-related signs or symptoms, as described above, or intolerable interferon-related side effects, including flu-like symptoms, chronic fatigue and/or musculoskeletal pain, depression, thyroid dysfunction, and autoimmune diseases that cannot be managed with supportive care approaches, are indicative of a need to consider a change in therapy [17].

### Sample patient 1—part 3

The patient was initiated on pegylated interferon at 90 μg weekly, which was poorly tolerated due to flu-like symptoms and arthralgias lasting 3 to 4 days after each injection for the first 4 weeks. The dose was reduced to 45 μg weekly, which was tolerated well and gradually increased to 135 μg weekly. After 9 months of interferon therapy, the patient experienced a slight decrease in phlebotomy requirements but continued splenomegaly, fatigue, and weight loss with no substantial change in WBC counts. In addition, his wife reported a withdrawn and dysphoric mood atypical for him, which is a known side effect of interferon therapy. Because of this, pegylated interferon was discontinued, and ruxolitinib was started at 10 mg twice daily (bid). This resulted in rapid improvement in mood symptoms, weight gain, and reduced splenomegaly.

### Sample patient 2—part 3

After demonstrating signs of HU intolerance, the patient was switched to ruxolitinib 1 year after diagnosis, with immediate resolution of mouth sores, continued control of headaches, and improvement in energy level. This also eliminated the need for phlebotomy.

## Second-line treatment

Ruxolitinib is my preferred second-line therapy for most patients who are resistant to or intolerant of first-line therapy [16, 30]. This recommendation is supported by multiple large, randomized, phase 3 clinical trials that demonstrated clinical benefit with ruxolitinib, including RESPONSE [22, 23, 29], RESPONSE-2 [39–41], and RELIEF [20].

The RESPONSE study consisted of patients who were unresponsive or intolerant to HU, had splenomegaly, and were phlebotomy-dependent. Patients treated with ruxolitinib were more likely to achieve hematocrit, WBC count, and platelet count control; reduction in spleen volume; reduction in symptoms; and lower phlebotomy rates compared with best available therapy (BAT) [22]. These responses were durable through 5 years of follow-up [29]. For patients originally randomized to BAT, those who crossed over to ruxolitinib experienced similar clinical benefit as those originally randomized to ruxolitinib [23]. Finally, a post hoc analysis from the RESPONSE trial that examined *JAK2*V617F allele burden demonstrated that patients who received ruxolitinib had greater reductions in allele burden compared with those receiving BAT [42]. Although the clinical relevance of these allele burden reductions is unclear, higher allele burden levels have been associated with more severe disease [43].

The RESPONSE-2 study evaluated ruxolitinib versus BAT in HU-resistant patients who were phlebotomy dependent but without splenomegaly. Ruxolitinib was superior to BAT at reducing phlebotomy requirement and achieving hematocrit, WBC count, and platelet count control. Patients treated with ruxolitinib also experienced better symptom control and improvements in QoL compared with BAT [40]. Similar to RESPONSE, these responses were also durable through 5 years of follow-up [41].

It is important to emphasize that ruxolitinib provided hematocrit control and improvements in PV-related symptoms regardless of the presence or absence of splenomegaly, indicating that splenomegaly should not be a prerequisite for initiating ruxolitinib treatment [22, 40].

Ruxolitinib has also been demonstrated to provide clinical benefit regardless of whether patients were previously treated with interferon or HU. In a post hoc analysis of patients who were treated with an interferon formulation as investigator-selected BAT in the RESPONSE and RESPONSE-2 studies, patients who crossed over to ruxolitinib experienced clinical improvements including hematologic and spleen response as well as reduced phlebotomy requirement [44].

Several studies have evaluated the effects of ruxolitinib on thrombotic events. In RESPONSE through week 32, thromboembolic events occurred in one patient receiving ruxolitinib and 6 patients receiving BAT [22]. Exposure-adjusted rates were also lower for patients receiving ruxolitinib than BAT (1.2 vs. 8.2 per 100 patient-years, respectively) through 5 years of follow-up [29]. Similarly, there was one thrombotic event in a patient receiving ruxolitinib and 3 for those receiving BAT in the primary RESPONSE-2 analysis [40]. Exposure-adjusted rates of any-grade thromboembolic events were 1.5 per 100 patient-years for patients receiving ruxolitinib and 3.7 for patients receiving BAT through 5 years of follow-up [39, 41]. Additionally, a meta-analysis covering 663 patients from RESPONSE, RESPONSE-2, RELIEF, and MAJIC reported similar rates of thrombotic events for ruxolitinib versus BAT (3.09 vs. 5.51; *P* = 0.98) [28]. Finally, a recent retrospective analysis evaluating real-world ruxolitinib treatment in patients with HU-resistant/intolerant PV demonstrated a significantly lower rate of arterial thrombosis with ruxolitinib versus BAT (0.4 vs. 2.3; *P* = 0.03) but no significant differences in venous thrombosis, major bleeding, or survival [45].

Although presence of new or worsening PV-related symptoms alone may be an indicator for treatment change, potential benefit may extend to only a subpopulation of patients. The phase 3b RELIEF study consisted of patients who had PV-related symptoms but were generally well controlled on a stable dose of HU. The study evaluated switching treatment to ruxolitinib versus remaining on HU. Among patients who switched to ruxolitinib, some experienced improvements in symptoms that contributed to a positive trend in symptom control at week 16 compared with HU; however, this did not meet statistical significance [20].

Although ruxolitinib is the preferred second-line treatment in my practice, interferon formulations (including ropeginterferon alfa-2b and pegylated interferon alfa-2a) are appropriate second-line treatment options for patients who are intolerant or have inadequate response to HU [30, 46].

In PROUD-PV and its extension study, CONTINUATION-PV, ropeginterferon alfa-2b was compared with HU in patients with early-stage PV with no history of cytoreductive treatment or less than 3 years of previous HU treatment of which they were resistant or intolerant. In PROUD-PV, noninferiority versus HU was not achieved at 1 year for the combined primary endpoint: hematologic response (defined as hematocrit < 45% with no phlebotomy in the past 3 months, platelet count < 400 × 10^9^/L, and leukocyte count < 10 × 10^9^/L) and normal spleen size [32]. However, response to ropeginterferon alfa-2b increased over time with improved hematologic response and improvement in disease burden compared with HU at 36 months in CONTINUATION-PV [32]. Ropeginterferon alfa-2b may also provide additional benefit in patients with low-risk PV, as assessed in the low-PV study. This trial compared ropeginterferon alfa-2b added to a standard phlebotomy regimen (including 100 mg aspirin daily) with phlebotomy alone in patients with low-risk PV. Hematocrit control was maintained in more patients receiving add-on ropeginterferon alfa-2b than phlebotomy alone [32, 47].

Additionally, a phase 2 study from the Myeloproliferative Disorders Research Consortium recently demonstrated a 60% overall response rate and 22% complete response among 50 patients with PV treated with pegylated interferon alfa-2a [33]. Statistically significant improvement in MPN-related symptoms was also observed for PV and ET patients combined; however, treatment-emergent adverse events may have offset these improvements, resulting in patient-reported measures of quality of life remaining stable [33].

Finally, rusfertide, a peptide mimetic of the master iron regulator hepcidin, is currently in development as a non-cytoreductive option in PV to reverse iron deficiency and achieve hematocrit control [48, 49]. Rusfertide demonstrated hematocrit control (< 45%) and eliminated the need for phlebotomy in two phase 2 studies in patients with PV [48, 49]. The safety and efficacy of rusfertide are also being investigated in the phase 3 randomized VERIFY study (NCT05210790).

### Sample patient 1—part 4

The ruxolitinib dose was subsequently increased to 20 mg bid after 9 months of treatment due to continued need for phlebotomies. To date, the patient has been receiving ruxolitinib for 4.5 years, with the dose remaining at 20 mg bid, with nonpalpable splenomegaly, no need for phlebotomies, and good QoL.

### Sample patient 2—part 4

The patient has been receiving ruxolitinib for 7 years, and her PV remains well controlled without need for phlebotomy, with good functional status and no headaches, although she struggles with weight gain that is likely attributable to ruxolitinib.

## Assessing second-line treatment safety

Patients receiving treatment with ruxolitinib should be monitored for certain adverse events that have been observed in short- and longer-term clinical trials in patients with PV. These include cytopenias, opportunistic infections, herpes zoster reactivation, nonmelanoma skin cancer, hypertension, and weight gain [20, 22, 23, 40]. One of the goals of treatment in PV is to lower elevated blood counts; as a result, cytopenias are the most common adverse events with ruxolitinib. These often present as grade 1 or 2, as observed in the RESPONSE and RESPONSE-2 trials [22, 40]. Although opportunistic infections do occur in patients treated with ruxolitinib, long-term follow-up in RESPONSE and RESPONSE-2 demonstrated that exposure-adjusted infection rates (excluding herpes zoster) were lower with ruxolitinib than with BAT [29, 41]. With respect to weight gain, one retrospective study of patients with myeloproliferative neoplasms treated with ruxolitinib found that > 50% of patients gained > 5% of their baseline body weight [50]. Physicians initiating treatment with ruxolitinib in patients with PV should consider monitoring these patients for metabolic effects (e.g., obesity, hyperglycemia, dyslipidemia, hypertension, hepatic steatosis) and providing dietary counseling or lifestyle management recommendations to help offset potential weight gain. Overall, adverse effects should be monitored closely and can be managed by dose reduction or interruption, as detailed in the next section.

For patients receiving ropeginterferon alfa-2b, results from the PROUD-PV and CONTINUATION-PV studies revealed that treatment-related toxicities overall occurred at similar rates as HU treatment. The most common adverse events with ropeginterferon alfa-2b were cytopenias (thrombocytopenia, leukopenia, and anemia; predominantly grade 1 or 2), liver laboratory abnormalities (increased γ-glutamyltransferase, alanine aminotransferase, and aspartate aminotransferase), and flu-like symptoms (fatigue, headache, dizziness).

Regarding rusfertide, although phase 3 studies are underway in PV, phase 2 studies have demonstrated a favorable safety profile, with most adverse events classified as grade 1 or 2. The most frequent adverse event was transient injection site reaction, with one study reporting its occurrence in 59% of patients [48, 49].

## Ruxolitinib dose optimization

**Fig. 2:**
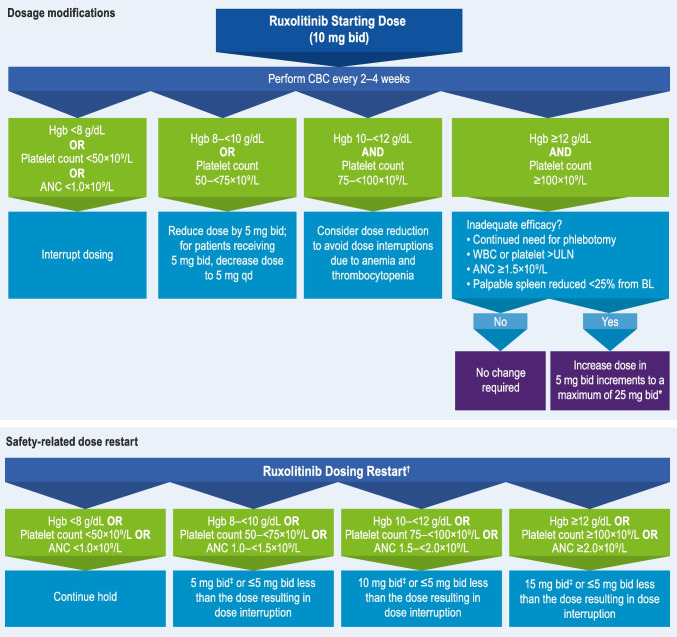
Dosage recommendations for ruxolitinib. *Not during the first 4 weeks of therapy or more frequently than every 2 weeks. ^†^Based on the most severe category of Hgb, platelet count, or ANC abnormality. ^‡^Continue treatment for ≥ 2 weeks; if dose stabilizes, it may be increased by 5 mg bid. ANC, absolute neutrophil count; bid, twice daily; BL, baseline; CBC, complete blood count; Hgb, hemoglobin; qd, once daily; ULN, upper limit of normal; WBC, white blood cell count

The recommended starting dose for patients with PV is 10 mg bid, and doses may be titrated based on observed safety and efficacy outcomes [24]. Guidelines for treatment interruption and restarting dosing, as well as dose modifications related to insufficient response to ruxolitinib, are included in Fig. 2.

Ruxolitinib dosage modifications have been evaluated in a real-world setting. A medical chart review of patients with PV who switched from HU to ruxolitinib found that only half initiated treatment at the recommended dose. Dose modifications were common (27%) in the first 6 months of ruxolitinib treatment, and most patients achieved hematocrit control and had extended treatment with ruxolitinib [51]. This information reinforces the importance of selecting the proper ruxolitinib starting dose and actively titrating the dose thereafter based on safety and efficacy observations to avoid treatment interruptions and maximize clinical benefit. Nevertheless, treatment interruption may be required for some patients, especially for cytopenias (Fig. 2) [24]. Although some patients with a related myeloproliferative neoplasm, myelofibrosis, who discontinued ruxolitinib experienced ruxolitinib discontinuation syndrome, which constitutes a rebound in myelofibrosis symptoms after treatment discontinuation [52], this has not been observed in patients with PV.

## Ropeginterferon alfa-2b dose optimization

The ropeginterferon alfa-2b recommended starting dose is 100 μg by subcutaneous injection every 2 weeks or 50 μg if receiving concomitant HU. Dosage should be increased by 50 μg every 2 weeks (up to a maximum of 500 μg) until target hematologic parameters are achieved. Dose interruption or discontinuation is recommended in response to certain adverse reactions such as liver enzyme elevation (with or without concomitant bilirubin elevation or other evidence of hepatic decompensation), cytopenias, or depression. Complete blood count should be monitored every 2 weeks when titrating and modifying dose. Phlebotomy may be used as needed to maintain safe hematocrit values. Following a treatment interruption and resolution of instigating factors, ropeginterferon alfa-2b should be restarted at the previously attained dose or reduced to the next lower dose level if drug-related toxicities arise. If efficacy is insufficient after a dose decrease, increase dose to the next higher dose level after recovery to grade 1 toxicity.

## Conclusion

Patients with high-risk PV often experience clinical benefit with first-line cytoreductive treatments; however, treatment efficacy may diminish over time, and treatment-related toxicities may be challenging for some patients. A change in treatment is often beneficial for this patient population, with ruxolitinib the current preferred second-line treatment option. Based on evidence from 3 large phase 3 clinical trials, ruxolitinib improves PV-related signs and symptoms in the second line, and long-term treatment is feasible with dose modifications. Additional second-line options are emerging with distinct therapeutic and toxicity profiles, which reinforce the need to establish clear, patient-specific treatment goals in order to provide individualized therapy.
