# Correction to: Treatment of hydroxyurea-resistant/intolerant polycythemia vera: a discussion of best practices

**DOI:** 10.1007/s00277-023-05235-0

**Published:** 2023-04-24

**Authors:** Andrew T. Kuykendall

**Affiliations:** Department of Malignant Hematology, H. Lee Moffitt Cancer Center, Tampa, FL 33612 USA

**Correction to: Annals of Hematology**

10.1007/s00277-023-05172-y

The article “Treatment of hydroxyurea-resistant/intolerant polycythemia vera: a discussion of best practices”, written by Andrew T. Kuykendall, was originally published electronically without open access. With the author(s)’ decision to opt for Open Choice the copyright of the article changed on 17 April 2023 to ©The Author(s) 2023 and the article is forthwith distributed under a Creative Commons Attribution 4.0 International License, which permits use, sharing, adaptation, distribution and reproduction in any medium or format, as long as you give appropriate credit to the original author(s) and the source, provide a link to the Creative Commons licence, and indicate if changes were made. The images or other third party material in this article are included in the article’s Creative Commons licence, unless indicated otherwise in a credit line to the material. If material is not included in the article’s Creative Commons licence and your intended use is not permitted by statutory regulation or exceeds the permitted use, you will need to obtain permission directly from the copyright holder. To view a copy of this licence, visit http://creativecommons.org/licenses/by/4.0.

The original article has been corrected.

